# Evolving Landscape of Systemic Therapy for Hepatocellular Carcinoma: From Targeted Therapy and Combination Strategies to Emerging Therapies

**DOI:** 10.3390/biomedicines14081735

**Published:** 2026-07-31

**Authors:** Hui Li, Junyu Zhou, Xu Zhu

**Affiliations:** 1 The Center of Biomedical Engineering, Institute of Advanced Clinical Medicine, Peking University, Beijing 100191, China; lh0413dd@163.com (H.L.); benjaminjhou@bjmu.edu.cn (J.Z.); 2 Key Laboratory of Carcinogenesis and Translational Research (Ministry of Education/Beijing), Peking University Cancer Hospital & Institute, Beijing 100142, China

**Keywords:** hepatocellular carcinoma, signaling pathways, targeted therapy, combination therapy, novel therapies

## Abstract

**Background**: Hepatocellular carcinoma (HCC) is the predominant histological subtype of primary liver cancer, and many patients with unresectable disease require systemic therapy. This narrative review asks how current systemic regimens should be selected and sequenced, how oncogenic pathways inform resistance and biomarker development, and which emerging platforms have sufficient evidence for clinical translation. **Methods**: We synthesized key oncogenic pathways, pivotal trials, major guidelines, and selected translational studies cited in this review. Randomized phase III evidence was prioritized for clinical claims, whereas observational, early-phase, and preclinical studies were used to define evidence gaps and emerging approaches. **Results**: Immune-based combinations have reshaped first-line treatment for advanced HCC. Treatment selection must account for liver function, performance status, bleeding risk, and contraindications to anti-VEGF or immune checkpoint therapy. Evidence for post-immunotherapy sequencing remains largely observational, and validated predictive biomarkers are scarce. **Conclusions**: Future progress requires prospective sequencing studies, biomarker-enriched trials, and rigorous early-phase evaluation of cell, antibody, gene-editing, viral, and nanodelivery platforms.

## 1. Introduction and Review Scope

HCC treatment is determined by tumor stage, liver functional reserve, and competing risks arising from cirrhosis. The expansion of immune-based combinations has increased first-line options but has also complicated regimen selection and post-progression sequencing. Mechanistic and emerging-therapy evidence is clinically useful only when it clarifies treatment sensitivity, resistance, or testable biomarkers.

This review addresses three questions: how stage, liver function, and contraindications shape systemic treatment selection and sequencing; which molecular pathways have established or investigational therapeutic relevance; and which emerging platforms have evidence sufficient to justify clinical testing. Clinical recommendations are anchored preferentially to randomized phase III trials and major guidelines. Observational, early-phase, and preclinical findings are explicitly identified to preserve the boundary between current practice and hypothesis-generating evidence.

### 1.1. BCLC-Based Treatment Framework and Contemporary Guidelines

The Barcelona Clinic Liver Cancer (BCLC) framework integrates tumor burden, liver function, performance status and treatment feasibility. It remains a useful starting point for multidisciplinary decision-making, while acknowledging that stage migration and patient-specific factors may justify a treatment beyond the default stage-based recommendation [1].

Patients with BCLC 0 or A disease are generally considered for resection, ablation, or transplantation. For BCLC B disease, transarterial chemoembolization (TACE) remains a standard option for appropriately selected patients. Systemic therapy should be considered when TACE is unsuitable or refractory. BCLC C disease is generally managed with systemic therapy, whereas BCLC D disease requires symptom-focused supportive care and goals-of-care discussions [1,2].

Recent AASLD, EASL, APASL, and China Liver Cancer (CNLC) guidance converges on immune-based combinations as preferred first-line options for eligible patients with unresectable HCC and preserved liver function. However, regional approvals, reimbursement, liver reserve, bleeding risk, and access to multidisciplinary care influence treatment selection. Guideline recommendations should therefore be interpreted in the context of local availability and individual risk–benefit assessment [2,3,4,5].

Table 1 summarizes the practical treatment considerations across BCLC stages, including the selection of locoregional or systemic therapy, the timely transition from TACE to systemic treatment, the choice between immune-based combinations and tyrosine kinase inhibitor (TKI)-based therapy, and the principles guiding treatment sequencing after disease progression. It also highlights key clinical restrictions, including liver reserve, portal hypertension, bleeding or anti-VEGF-related risk, autoimmune disease, transplantation history, treatment tolerability, and local drug availability.

### 1.2. Patient Selection for Antibody-Based Immunotherapy Combinations

Antibody-based immunotherapy combinations should be selected after careful assessment of liver function, Eastern Cooperative Oncology Group performance status, tumor burden, vascular invasion, extrahepatic spread, and the competing risks associated with cirrhosis. The trial populations that established current first-line regimens largely had preserved liver function and good performance status; therefore, extrapolation to patients with Child–Pugh B liver function remains uncertain, and treatment should be individualized [2,3,4].

For atezolizumab plus bevacizumab, bleeding risk and suitability for anti-vascular endothelial growth factor therapy require particular attention. Upper gastrointestinal endoscopy and appropriate management of high-risk varices should be considered before initiating atezolizumab plus bevacizumab, particularly in patients with cirrhosis or portal hypertension. Active or uncontrolled autoimmune disease and prior solid-organ transplantation may limit the use of immune checkpoint inhibitor-based therapy because of the risk of immune-related complications. For bevacizumab-containing regimens, clinically significant proteinuria, uncontrolled hypertension, and substantial haemorrhagic risk should also be carefully assessed and may favor alternative systemic strategies. These factors primarily guide treatment safety and feasibility, but they are not validated predictive biomarkers of therapeutic efficacy [2,3].

### 1.3. Treatment Sequencing After First-Line Immunotherapy-Based Combinations

Treatment sequencing after first-line immune-based therapy remains an important evidence gap. Tyrosine kinase inhibitors, particularly lenvatinib or sorafenib, are commonly used in clinical practice after progression on or intolerance to atezolizumab plus bevacizumab. Cabozantinib and ramucirumab in patients with alpha-fetoprotein levels ≥ 400 ng/mL represent additional evidence-based options in their approved settings, although their pivotal trials were conducted predominantly in patients previously treated with sorafenib rather than after first-line immunotherapy [2,3,4].

Observational studies suggest that lenvatinib may be associated with favorable progression-free survival compared with sorafenib after atezolizumab plus bevacizumab. However, these comparisons are susceptible to confounding, selection bias, and regional differences in clinical practice, and should not be interpreted as establishing a universally preferred treatment sequence [6,7].

After durvalumab plus tremelimumab, TKI-based therapy is also a practical subsequent option, although direct prospective evidence defining the optimal sequence remains limited. Rechallenge with an immune checkpoint inhibitor or continuation of combination therapy beyond progression should currently be reserved for clinical trials whenever possible. At each treatment transition, therapeutic decisions should be individualized according to the reason for first-line discontinuation, residual liver function, prior adverse events, tumor kinetics, comorbidities, and patient preferences and goals [2,3,4,5].

**Figure 1:**
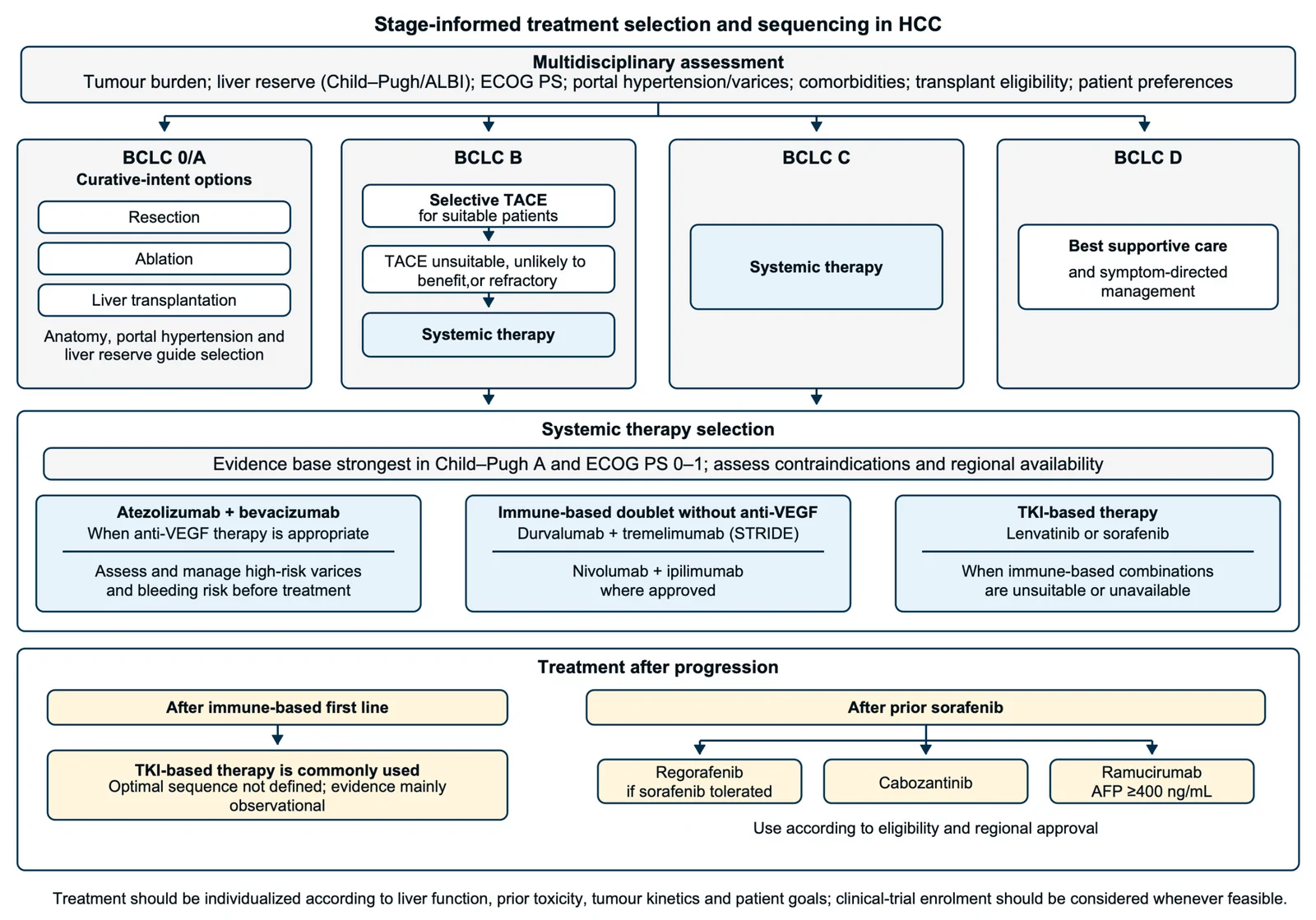
Framework for stage-informed treatment selection and systemic therapy sequencing in HCC.

These clinical considerations are integrated into a practical decision framework for unresectable HCC, linking BCLC stage, liver function, performance status, bleeding and immune-related risks, first-line regimen selection, and subsequent treatment options (Figure 1). The clinical uncertainties outlined above arise partly from tumor heterogeneity, adaptive signaling, and immune exclusion. The next section therefore examines molecular pathways only to the extent that they explain the activity, limitations, or resistance patterns of systemic therapies and identify testable biomarkers.

## 2. Key Signaling Pathways and Target Landscape

**Figure 2:**
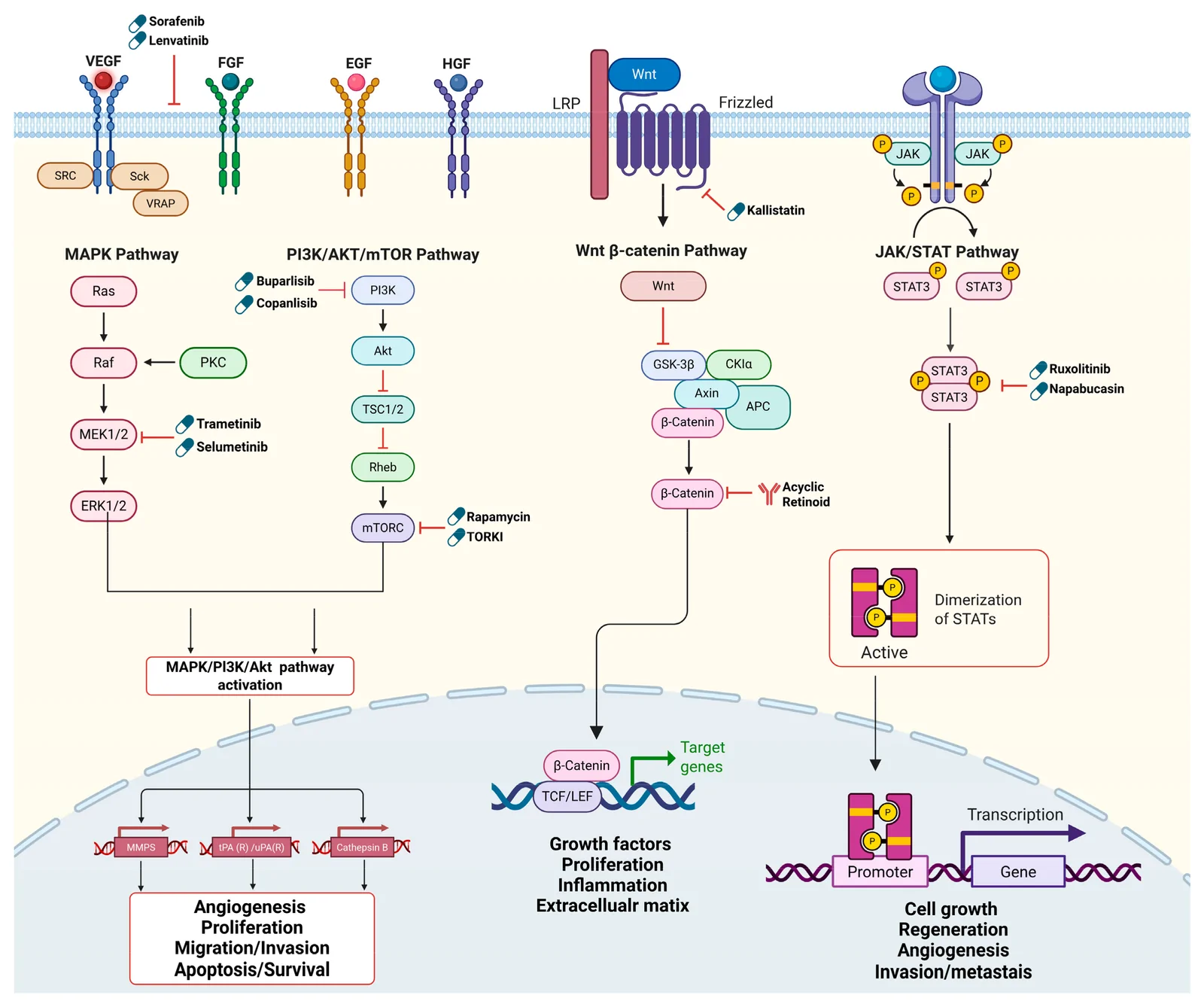
Key signaling pathways and therapeutic targets in HCC. Schematic representation of the MAPK, PI3K/AKT/mTOR, Wnt/β-catenin and JAK/STAT pathways; representative inhibitors and downstream processes are shown. Created in BioRender. Li, H. (2026) https://BioRender.com/qs0lcut (accessed on 3 July 2026).

Mechanistic understanding is most useful when it clarifies why established agents work, why resistance emerges, and which candidate targets remain unvalidated. In HCC, VEGF/VEGFR signaling has clear clinical relevance because it is engaged by approved multikinase inhibitors and antibody-based combinations. By contrast, direct targeting of the RAS/RAF/MEK/ERK, PI3K/AKT/mTOR, Wnt/β-catenin, and JAK/STAT pathways remains largely investigational. These pathways nevertheless provide a framework for interpreting tumor heterogeneity, immune exclusion, and adaptive resistance [8,9,10,11,12,13,14,15,16]. Figure 2 summarizes the principal networks and representative therapeutic targets.

### 2.1. Receptor Tyrosine Kinases Pathway

As central signaling hubs governing the malignant phenotype of HCC, receptor tyrosine kinases (RTKs) undergo ligand-induced autophosphorylation and subsequent activation of their intracellular kinase domains, thereby recruiting downstream adaptor proteins to form dynamic signaling complexes and initiate cascade activation of pathways such as the mitogen-activated protein kinase (MAPK) and PI3K/AKT pathways [17]. In HCC, aberrant activation of RTK family members exhibits distinct functional roles. Vascular endothelial growth factor receptor (VEGFR) primarily mediates pathological angiogenesis and remodeling of the tumor vascular microenvironment [18,19,20]. Fibroblast growth factor receptor (FGFR) and epidermal growth factor receptor (EGFR) sustain tumor cell proliferation and survival through activation of mitogenic signaling and suppression of apoptotic pathways [21,22,23]. Hepatocyte growth factor receptor (HGFR), also known as c-MET, promotes invasion and metastasis by inducing epithelial–mesenchymal transition (EMT) [24]. In addition, platelet-derived growth factor receptor (PDGFR) and insulin-like growth factor receptor (IGFR) facilitate tumor progression through stromal interactions and metabolic reprogramming, respectively [25,26,27]. Collectively, dysregulation of this multidimensional RTK signaling network drives the acquisition of malignant phenotypes, including uncontrolled proliferation, resistance to apoptosis, migration, and invasion.

In current clinical practice for HCC, RTK-related targets exhibit distinct therapeutic relevance. Among them, the VEGF/VEGFR-centered angiogenic pathway has become a fundamental component of systemic therapy for advanced HCC. Multikinase inhibitors simultaneously targeting VEGFR, FGFR, PDGFR, and related kinases have established robust evidence-based efficacy in both first-line and second-line treatment settings. Aberrant activation of the FGF19/FGFR4 axis is recognized as a critical driver of hepatocarcinogenesis, closely associated with specific molecular subtypes of HCC, enhanced tumor aggressiveness, and the development of therapeutic resistance. In parallel, the hepatocyte growth factor receptor c-MET markedly promotes tumor invasion and metastasis through induction of epithelial–mesenchymal transition (EMT), while activation of the MET signaling pathway has also been identified as a key mechanism underlying acquired resistance to agents such as sorafenib.

By contrast, the clinical translation of several other RTK family members, including EGFR and IGFR, remains challenging. For instance, the anti-EGFR monoclonal antibody cetuximab has demonstrated limited efficacy in HCC, potentially owing to compensatory activation of alternative signaling pathways. IGFR signaling primarily facilitates tumor progression through regulation of cellular metabolism and anti-apoptotic pathways; however, therapeutic strategies targeting this pathway largely remain at the stage of preclinical investigation or early-phase clinical evaluation, and no standard treatment paradigm has yet been established [15,28,29].

### 2.2. RAS/RAF/MEK/ERK Pathway

The RAS/RAF/MEK/ERK signaling pathway, a core driver of tumor initiation and progression, plays a central role in tumorigenesis, malignant transformation, and metastasis by regulating cell cycle progression, apoptosis resistance, and invadopodia formation [30,31,32]. Accumulating evidence indicates that aberrant activation of the RAS/RAF/MEK/ERK pathway is a key mechanism underlying HCC development. Ito et al. found that 58% of patients with HCC exhibited ERK pathway activation and upregulation of related genes [33]. Hoffmann et al. demonstrated that 33% of HCC cases harbored aberrant RAS mRNA expression, while MEK, ERK, and MAPK mRNAs were overexpressed in 40%, 50%, and 50% of HCC patients, respectively [34]. At the protein level, Liao et al. reported that immunohistochemistry showed positive H-ras protein expression in approximately 93.8% of HCC samples [35]; another study revealed significantly upregulated protein expression of RAS and its downstream effectors MEK and ERK in cirrhotic and HCC tissues, accompanied by aberrant activation of MAPK [36]. Gollob et al. found that RAF-1 protein was overexpressed in 91.2% of cirrhotic tissues and all HCC tissue samples, with higher expression intensity in HCC than in cirrhosis [37].

First-generation MEK inhibitors (e.g., CI-1040) inhibit MEK activity by non-competitively binding to an allosteric site in the catalytic domain. In combination with sorafenib, they synergistically suppress ERK phosphorylation and cell proliferation in HCC cell lines, and exhibit superior antitumor efficacy compared with either agent alone in nude mouse xenograft models [38]. A phase II trial of the MEK1/2 inhibitor selumetinib (AZD6244) showed that selumetinib reduced ERK phosphorylation levels in patients with HCC, but its monotherapy antitumor activity was modest [39]. The second-generation MEK inhibitor PD0325901 has approximately 500-fold greater inhibitory potency against MEK than CI-1040 and demonstrates strong antitumor activity in multiple tumor models [40]. The selective MEK1/2 inhibitor trametinib improves overall survival (OS) and progression-free survival (PFS) in patients with BRAF V600E/K-mutant melanoma; however, its efficacy in HCC remains to be further validated. To date, most MEK inhibitors have shown insufficient antitumor activity and limited clinical benefit in HCC [41]. Combination strategies of MEK inhibitors with EGFR monoclonal antibodies, targeted agents, or immunotherapies are still under investigation. In the future, biomarker-driven patient stratification may help overcome tumor heterogeneity and resistance mechanisms.

### 2.3. PI3K/AKT/mTOR Signaling Pathway

The PI3K/AKT/mTOR signaling pathway is frequently activated across a wide spectrum of malignancies [42]. Aberrant activation of the PI3K/AKT/mTOR axis has been identified in approximately 50% of patients with HCC [43], with its oncogenic functions primarily reflected in the following aspects: (1) metabolic reprogramming, in which mechanistic target of rapamycin complex 1 (mTORC1) activates sterol regulatory element-binding protein 1 (SREBP-1) through phosphorylation of serine/arginine-rich protein kinase 2 (SRPK2), thereby driving de novo lipogenesis [44]; (2) enhanced hypoxic adaptation, whereby hypoxia-inducible factor-2α (HIF-2α) upregulates the expression of the PI3K catalytic subunit p110β and promotes glycolysis and fatty acid oxidation via the AKT/mTORC1 axis [45]; (3) maintenance of invasive phenotypes, as aberrant expression of 3-phosphoinositide-dependent protein kinase-1 (PDK1) induces the formation of disseminated HCC lesions [46]; and (4) dysregulated autophagy, in which suppressor of cytokine signaling 5 (SOCS5) enhances the adhesive and invasive capacities of HCC cells by blocking mTORC1-mediated autophagosome–lysosome maturation [47].

PI3K inhibitors can generally be categorized into three major classes: pan-PI3K inhibitors, isoform-selective PI3K inhibitors, and dual PI3K/mTOR inhibitors [48,49]. Among these, the pan-PI3K inhibitor buparlisib (BKM120), which targets all class I PI3K isoforms, has previously demonstrated clinical benefit when combined with endocrine therapy in advanced breast cancer [50]. In HCC-related studies, both in vitro and in vivo experiments have shown that buparlisib exerts antitumor activity by inhibiting tumor cell proliferation and inducing apoptosis. However, owing to adverse events such as rash and hyperglycemia, current investigations remain largely confined to the preclinical stage [51,52]. Another pan-PI3K inhibitor, copanlisib (BAY80-6946), has been approved by the United States Food and Drug Administration for the treatment of follicular lymphoma, whereas its application in HCC is still under early-stage investigation [53,54].

Inhibitors targeting mTOR represent one of the earliest classes of agents developed against the PI3K/AKT/mTOR signaling pathway. First-generation mTOR inhibitors, including rapamycin and its analogs, suppress HCC growth through inhibition of mTORC1 phosphorylation; however, their long-term efficacy may be compromised by feedback activation of the IGF-IR/AKT signaling pathway [55,56,57]. Second-generation mTOR inhibitors simultaneously target both mTORC1 and mTORC2, thereby further suppressing AKT phosphorylation and enhancing pathway inhibition, although high rates of acquired resistance remain a major limitation [58]. Third-generation mTOR inhibitors, exemplified by Rapalink, combine allosteric and ATP-competitive inhibitory mechanisms, and have been shown to overcome resistance to earlier-generation agents in selected experimental models, exhibiting superior antitumor activity [59].

It should be noted that most studies investigating PI3K/AKT/mTOR pathway inhibitors in HCC remain at the preclinical or early-phase clinical stage, and high-level evidence demonstrating a definitive overall survival benefit is still lacking. Consequently, these agents have not yet been incorporated into standard systemic treatment strategies for HCC. Emerging evidence suggests that extensive crosstalk exists between the PI3K/AKT/mTOR pathway and other oncogenic signaling networks, including RTKs and MAPK pathways. Inhibition of a single signaling axis may therefore trigger compensatory pathway activation, which likely represents a major factor limiting the clinical efficacy of these targeted therapies. Future therapeutic strategies may require integration of molecular subtyping, biomarker-guided patient selection, and rational combination regimens to further enhance the clinical utility of targeting the PI3K/AKT/mTOR pathway.

### 2.4. The Wnt/β-Catenin Pathway

As a highly conserved developmental regulatory pathway, the Wnt/β-catenin signaling system plays a dual role in tissue homeostasis maintenance and malignant transformation. Aberrant activation of this pathway has been closely linked to the development and progression of various solid tumors [60,61]. In HCC, somatic mutations of the CTNNB1 gene (encoding β-catenin protein) occur in 20–40% of cases. These mutations enhance β-catenin protein stability by preventing β-catenin’s phosphorylation-dependent degradation, leading to persistent activation of downstream oncogenic transcriptional programs [62]. Of note, the spatiotemporal distribution of β-catenin is closely related to its pro-tumorigenic functions. In early-stage HCC, β-catenin, as part of the adherens junction complex with cadherins (e.g., E-cadherin), amplifies growth factor receptor (e.g., EGFR) signaling cascades [63]. As the disease progresses, nuclear accumulation of β-catenin significantly upregulates matrix metalloproteinase expression, thereby promoting HCC invasion and metastasis [64].

Small-molecule inhibitors targeting the Wnt/β-catenin pathway suppress HCC cell proliferation and induce apoptosis by competitively binding to receptors or blocking the protein–protein interaction between β-catenin and T-cell factor [65]. The pan-Frizzled monoclonal antibody OMP-18R5 targets extracellular domains or membrane-associated proteins to inhibit ligand-receptor signaling while indirectly exerting antitumor effects by activating the innate immune system [66]. Additionally, kallistatin blocks Wnt signaling and suppresses angiogenesis by antagonizing LRP6 [67]. Acyclic retinoids, as synthetic vitamin A derivatives, inhibit Wnt/β-catenin pathway activity by downregulating proto-oncogene expression [68]. Studies have also shown that cytochrome P450 2E1 (CYP2E1) negatively regulates β-catenin signaling through a GSK-3β-independent pathway, thereby inhibiting HCC cell activity [69].

At present, the clinical druggability of the Wnt/β-catenin pathway in HCC remains limited. Most agents targeting this pathway are still in preclinical or early-stage clinical investigation, and high-level evidence demonstrating clear survival benefits is lacking. Recent studies suggest that the more important clinical significance of aberrant Wnt/β-catenin pathway activation may lie in its close association with tumor immune microenvironment remodeling and resistance to immunotherapy. β-catenin activation can inhibit dendritic cell recruitment and CD8+ T cell infiltration, creating an “immune-cold tumor” phenotype and thereby reducing sensitivity to immune checkpoint inhibitors (ICIs). This mechanism has been linked to primary resistance to anti-PD-1/PD-L1 therapy in some patients. Thus, aberrant Wnt/β-catenin status has been recognized as a potential predictive biomarker for immunotherapy, providing a rationale for combining Wnt-targeted therapy with ICIs. Future studies should further investigate the crosstalk between the Wnt/β-catenin pathway and the tumor immune microenvironment, metabolic reprogramming, and resistance networks, and establish a precise stratification system to optimize combination immunotherapy strategies and identify patients most likely to benefit.

### 2.5. The JAK/STAT Pathway

The Janus kinase (JAK)/signal transducer and activator of transcription (STAT) pathway comprises JAK1–3, tyrosine kinase 2 (TYK2), and downstream transcriptional regulators STAT1-6. Its core functions include regulation of cell proliferation, stemness maintenance, and immune microenvironment remodeling. STAT family members exhibit functional polarization in HCC. (1) Pro-tumorigenic effects: STAT3 promotes the expression of anti-apoptotic genes and participates in the progression of various malignancies [70]. As the most abundantly expressed effector molecule of the JAK/STAT pathway, aberrant STAT3 expression in HCC tissues has been associated with shortened patient survival and increased risk of recurrence [71,72]. (2) Tumor-suppressive functions: In HCC, STAT1 predominantly exists in its unphosphorylated form, whereas phosphorylated STAT1 exerts antitumor activity [73]. The activity of STAT1 in HCC is influenced by VEGF expression, suggesting that the antitumor effect of STAT1 may be linked to inhibition of angiogenesis [74]. In addition, studies have confirmed that STAT2 exerts antitumor effects in HCC by suppressing oncogene transcription [75].

First-generation pan-JAK inhibitors broadly inhibit JAK family members. Tofacitinib was developed for immune-mediated and transplant-related indications, but its current US approvals are limited to specified inflammatory diseases and do not include organ-transplant rejection [76,77]. Baricitinib, a selective JAK1/2 inhibitor, is used for rheumatoid arthritis [78]. Investigation of these agents in HCC remains largely mechanistic or preclinical. Ruxolitinib has inhibited invasion and metastasis in HCCLM3 cells [79], while pacritinib and STAT3-directed agents have shown preclinical activity [80,81]. TYK2 and dual TYK2/JAK1 inhibitors remain investigational [82].

Most JAK/STAT inhibitors have not yet demonstrated clear clinical benefit in HCC, possibly due to the dual biological effects of this pathway in promoting pro-tumorigenic inflammation and mediating anti-tumor immunity. Since HCC mostly develops in the context of chronic liver disease and cirrhosis, the JAK/STAT pathway is involved in both pro-tumorigenic inflammatory responses and antitumor immune activation. How to avoid compromising immune surveillance while suppressing tumor progression represents a key challenge for the clinical translation of targeting this pathway. In addition, biomarker screening for subpopulations with aberrant JAK/STAT activation, as well as the synergistic mechanisms of combining JAK/STAT inhibitors with immune checkpoint inhibitors or anti-angiogenic therapy, may become important directions for optimizing HCC treatment in the future.

Across these pathways, clinical maturity is uneven. VEGF/VEGFR signaling is linked to approved multikinase inhibitors and anti-angiogenic combinations; MAPK and PI3K/AKT/mTOR inhibition has not produced a standard HCC therapy; Wnt/β-catenin is more relevant as a candidate marker of immune exclusion than as a validated drug target; and JAK/STAT inhibition remains preclinical or early phase. This evidence hierarchy provides a framework for interpreting the agent-specific trials discussed next.

## 3. Classification and Clinical Evidence of Molecular Targeted Therapy for HCC

Clinical evidence for targeted monotherapy is concentrated in anti-angiogenic multikinase inhibitors and the biomarker-selected use of ramucirumab. Agents directed at c-MET, FGFR4, mTOR, and other pathway-specific targets have generally produced negative phase III results or remain in early development. This section therefore distinguishes established survival evidence from exploratory biological rationale and early-phase signals.

### 3.1. Multi-Target Tyrosine Kinase Inhibitors

#### 3.1.1. Sorafenib

As the first targeted agent approved for first-line treatment of advanced HCC, sorafenib blocks tumor cell proliferation and angiogenesis by inhibiting multiple kinases, including RAF, VEGFR, and PDGFR [13]. The multicenter phase III SHARP trial demonstrated a median overall survival (mOS) of 10.7 months in the sorafenib group versus 7.9 months in the placebo group, with 1-year survival rates of 44% and 33%, respectively [83].

#### 3.1.2. Lenvatinib

Lenvatinib potently and selectively inhibits VEGFR1–3, FGFR1–4, PDGFRα, RET, and KIT [28]. The phase III REFLECT trial, which performed a head-to-head comparison with sorafenib, showed that lenvatinib met the non-inferiority endpoint for first-line treatment of advanced HCC, with an mOS of 13.6 months versus 12.3 months for sorafenib [84].

#### 3.1.3. Donafenib

Donafenib is a deuterated derivative of sorafenib that targets VEGFR, PDGFR, and RAF kinases [85]. In the randomized, open-label phase II–III ZGDH3 trial conducted at 37 centers in China, donafenib achieved a median overall survival of 12.1 months versus 10.3 months with sorafenib and a lower incidence of grade ≥ 3 adverse events (38% vs. 50%) [86].

#### 3.1.4. Regorafenib

Regorafenib has a target profile similar to that of sorafenib, with more potent inhibition of VEGFR2 and TIE2. It is the standard second-line therapy after disease progression on sorafenib [87]. The RESORCE trial reported an mOS of 10.6 months for regorafenib versus 7.8 months for placebo [14].

### 3.2. Angiogenesis Inhibitors

#### 3.2.1. Bevacizumab

As a representative anti-angiogenic agent, bevacizumab blocks the binding of VEGF to its receptor and reduces aberrant vascular formation [88]. In the IMbrave150 trial, bevacizumab in combination with atezolizumab as first-line therapy for HCC achieved a median overall survival (mOS) of 19.2 months, representing a 5.5-month improvement over the sorafenib arm, and increased the objective response rate (ORR) to 35% [89].

#### 3.2.2. Ramucirumab

Ramucirumab exerts antitumor activity by targeting the extracellular domain of the VEGFR-2 [90]. REACH-2 was the first phase III study to show positive results in a biomarker-selected population of patients with HCC [91]. In patients with advanced HCC who had received prior first-line sorafenib and had baseline alpha-fetoprotein (AFP) ≥ 400 ng/mL, ramucirumab prolonged overall survival by 1.2 months compared with placebo, offering a new treatment option for patients with elevated baseline AFP.

### 3.3. c-MET Inhibitors

#### 3.3.1. Cabozantinib

c-MET is the receptor tyrosine kinase for hepatocyte growth factor (HGF). Aberrant activation of c-MET may promote tumor cell proliferation, migration, and epithelial–mesenchymal transition (EMT) through the PI3K/AKT/mTOR and RAS/MAPK pathways. In addition, c-MET can interact with VEGFR2 to induce tumor angiogenesis [92,93]. Cabozantinib is an oral small-molecule multi-kinase inhibitor targeting VEGFR2, MET, and RET. In the CELESTIAL trial, cabozantinib as second-line therapy for advanced HCC achieved a median overall survival (mOS) of 10.2 months versus 8.0 months in the placebo group, and a median progression-free survival (mPFS) of 5.2 months versus 1.9 months in the placebo group [94].

#### 3.3.2. Tivantinib

Tivantinib is an oral, non-selective c-MET tyrosine kinase inhibitor. A phase II trial (NCT00988741) showed only modest improvements in median time to progression (mTTP) and OS in patients with advanced HCC (1.6 months vs. 1.4 months; 6.6 months vs. 6.2 months, respectively). However, significantly better efficacy was observed in the subgroup with high c-MET expression (mTTP 2.7 months vs. 1.4 months), with a favorable safety profile [95]. A subsequent phase III study (NCT01755767) further found that although the mOS in the tivantinib monotherapy arm was 8.4 months, it was not significantly different from that in the placebo arm (9.1 months; HR = 0.97, *p* = 0.81). These results suggest that while tivantinib failed to significantly improve survival, biomarker-stratified strategies based on c-MET expression levels remain feasible [96].

### 3.4. FGFR Inhibitors

#### 3.4.1. Fisogatinib (BLU-554)

Aberrant signaling of the FGF/FGFR pathway is closely associated with the development of various malignancies. In HCC, activation of the FGF19/FGFR4 axis drives the Wnt/β-catenin pathway and promotes tumor proliferation and metabolic reprogramming [97]. Kim et al. demonstrated that aberrant activation of the FGF19/FGFR4 signaling pathway promotes the transformation of liver lesions into HCC by regulating bile acid metabolism, enhancing cell proliferation, and inhibiting apoptosis [98]. Fisogatinib is a highly selective FGFR4 inhibitor targeting patients with advanced HCC harboring FGF19 amplification or high expression. Ongoing phase I trial data have shown promising clinical benefits of BLU-554 in patients with advanced HCC and aberrant FGF19-FGFR4 expression [99].

#### 3.4.2. Brivanib

Brivanib simultaneously inhibits FGFR-1, FGFR-2, FGFR-3, VEGFR-2, and VEGFR-3. Completed phase I and II trials have confirmed the efficacy and safety of brivanib in the treatment of HCC [100,101,102]. However, phase III studies were prematurely terminated because survival benefit did not surpass that of sorafenib [103,104].

### 3.5. mTOR Inhibitors

#### 3.5.1. Everolimus

mTOR is a core effector molecule of the PI3K/AKT pathway and is aberrantly activated in 40–50% of HCC cases [105,106]. Studies have suggested that mTOR may be involved in aberrant mutations of the p53 and Tsc1 genes in HCC cells [107]. Everolimus, an oral mTOR inhibitor, demonstrated a median overall survival (mOS) of 7.5 months and a disease control rate (DCR) of 44% as monotherapy for advanced HCC in a phase II study [108]. However, in the subsequent phase III EVOLVE-1 trial (NCT01035229), no significant differences were observed in mOS (7.6 months vs. 7.3 months) or time to progression (TTP; 3.0 months vs. 2.6 months) compared with the control arm. Although everolimus had a better safety profile, no survival benefit was confirmed [109].

#### 3.5.2. Sirolimus and Temsirolimus

In addition to inhibiting mTOR, sirolimus blocks VEGF secretion and its downstream signaling. A small phase II trial reported an mOS of 6.6 months and an objective response rate (ORR) of 8% for sirolimus monotherapy in advanced HCC [110]. Its derivative temsirolimus (approved for renal cell carcinoma) achieved an mOS of 8.9 months in a phase II study of HCC; however, the primary endpoint of progression-free survival (PFS) was only 2.8 months, falling short of expectations [111].

Taken together, the clinical evidence for targeted monotherapy in HCC is concen-trated in a relatively limited group of agents with phase III survival benefit, principally multikinase inhibitors and biomarker-selected ramucirumab, whereas several path-way-directed agents have failed to improve outcomes in late-stage trials. Table 2 con-solidates the major efficacy data to facilitate comparison of therapeutic targets, control regimens, and median overall survival. The modest and heterogeneous benefits achieved with single-agent approaches also provide a clear clinical rationale for the combination strategies discussed in the following section.

## 4. Advances in Combination Therapy for HCC

Combination regimens in HCC should be interpreted according to their components and level of evidence. Phase III data support immune checkpoint blockade combined with anti-angiogenic therapy and dual immune checkpoint inhibition in selected first-line populations. Locoregional-systemic combinations require separate interpretation because disease stage, tumor distribution, and study design vary substantially.

The subsections below distinguish randomized phase III evidence from single-arm and retrospective signals, and separate targeted-plus-immunotherapy regimens from dual immune checkpoint blockade and locoregional-systemic strategies.

### 4.1. Targeted Therapy Combined with Immunotherapy

#### 4.1.1. IMbrave150

The IMbrave150 trial was the first phase III study to demonstrate superior survival benefit of an immunotherapy-based combination over sorafenib in advanced HCC. The combination of atezolizumab plus bevacizumab (“T + A” regimen) significantly improved overall survival (OS; 19.2 vs. 13.4 months) and progression-free survival (PFS; 6.9 vs. 4.3 months) compared with sorafenib. In the updated analysis, treatment-related grade 3–4 adverse events occurred in 43% of patients receiving atezolizumab plus bevacizumab and 46% of those receiving sorafenib, indicating a manageable safety profile [89].

#### 4.1.2. ORIENT-32

ORIENT-32 was a randomized, open-label phase II–III study conducted at 50 clinical sites in China among patients with HBV-associated unresectable HCC. Median PFS was 4.6 months with sintilimab plus the bevacizumab biosimilar IBI305 versus 2.8 months with sorafenib; the combination reduced the risk of death by 43% (HR, 0.57), with a 1-year OS rate of 62.4% in the sintilimab–IBI305 group. ORIENT-32 provided phase III evidence supporting an anti-PD-1 plus anti-VEGF antibody combination in the first-line setting, although the exclusively Chinese, HBV-dominant population should be considered when generalizing these findings [112].

#### 4.1.3. KEYNOTE-524

The KEYNOTE-524 trial was an open-label, single-arm phase Ib study evaluating the efficacy and safety of pembrolizumab combined with lenvatinib as first-line therapy for patients with advanced unresectable HCC. The objective response rate (ORR) per modified Response Evaluation Criteria in Solid Tumors (mRECIST) was 46%, with a median duration of response (DOR) of 8.6 months, a disease control rate (DCR) of 88%, and a median overall survival (mOS) of 22.0 months. Of note, KEYNOTE-524 lacked a standard-of-care control arm, and its efficacy results may be influenced by baseline patient characteristics, selection of the enrolled population, and assessment criteria. The observed high response rate may not necessarily translate into stable long-term survival benefit, and further phase III trials are needed to confirm its efficacy [113].

#### 4.1.4. LEAP-002

The LEAP-002 study was the first phase III trial to use lenvatinib as the control arm, evaluating the efficacy and safety of lenvatinib plus pembrolizumab versus lenvatinib plus placebo as first-line therapy for HCC. The results showed that the mOS difference did not reach statistical significance, but subgroup analyses suggested potential benefit in certain patients (e.g., those with AFP > 400 ng/mL) [114]. In the aforementioned KEYNOTE-524 study, lenvatinib plus pembrolizumab showed promising efficacy; however, in LEAP-002, the combination did not meet the dual primary endpoints. Possible reasons include the following: LEAP-002 adopted a double-blind design, whereas KEYNOTE-524 was an open-label trial; the control arm of LEAP-002 used lenvatinib monotherapy, which has an ORR significantly superior to sorafenib, thereby increasing the statistical power requirements of the trial design; additionally, differences in baseline patient characteristics, treatment duration, and assessment criteria between the two studies may have contributed to the discrepant results.

#### 4.1.5. COSMIC-312

COSMIC-312 evaluated the efficacy and safety of cabozantinib plus atezolizumab versus sorafenib as first-line treatment for advanced HCC [115]. The study demonstrated a significant improvement in progression-free survival (PFS) with the combination regimen compared with sorafenib (6.8 vs. 4.2 months). However, overall survival (OS) did not significantly differ between the two treatment arms, and no new safety signals were observed. Subgroup analyses suggested that Asian patients derived greater PFS benefit from cabozantinib plus atezolizumab, with trends toward improved PFS and OS also observed in patients with HBV-related HCC.

Nevertheless, the reasons underlying the lack of a clear OS benefit in COSMIC-312 remain uncertain. Compared with IMbrave150, COSMIC-312 included a lower proportion of Asian patients and HBV-related HCC cases, which may have influenced therapeutic responsiveness given the biological heterogeneity of HCC across different etiologies and geographical regions. In addition, multiple factors—including trial design, post-progression therapies, improved outcomes in the control arm, and the limited therapeutic margin of the combination regimen—may also have contributed to the negative OS outcome. Further prospective studies are therefore warranted to clarify the optimal patient populations and combination strategies according to etiological background and regional characteristic.

#### 4.1.6. HEPATORCH

In the randomized phase III HEPATORCH trial, toripalimab plus bevacizumab improved progression-free and overall survival compared with sorafenib in previously untreated advanced HCC. The regimen has been approved in China. Cross-trial differences in populations and design preclude direct ranking against other first-line combinations [116].

### 4.2. Dual Immune Checkpoint Inhibitor Combination Therapy

#### 4.2.1. HIMALAYA

The HIMALAYA study is the first global phase III trial to demonstrate positive results for a dual immune checkpoint inhibitor combination in advanced HCC, marking the advent of first-line dual immunotherapy for HCC. The study adopted the STRIDE (Single Tremelimumab Regular Interval Durvalumab) regimen, consisting of a single high dose of the CTLA-4 inhibitor tremelimumab combined with regular, sustained use of the PD-L1 inhibitor durvalumab. This regimen is designed to enhance the initial T-cell response through short-course CTLA-4-driven immune activation while mitigating the immune-related toxicities typically associated with long-term conventional dual immunotherapy. The results showed that the STRIDE regimen significantly improved overall survival (OS) compared with sorafenib, with a generally manageable safety profile. Five-year follow-up data presented at the 2024 ESMO Asia Congress showed that the 5-year OS rate in the STRIDE arm was 19.6%, approximately double that of the sorafenib arm (9.4%), suggesting that some patients may achieve long-term survival benefit [117].

Unlike combination regimens represented by atezolizumab plus bevacizumab, the STRIDE regimen does not rely on anti-VEGF therapy, offering important clinical value for patients with contraindications to anti-angiogenic therapy. For patients who are unsuitable for anti-VEGF agents due to conditions such as esophagogastric varices, high bleeding risk, severe proteinuria, or cardiovascular disease, the STRIDE regimen provides a new optional strategy for first-line systemic therapy in advanced HCC. Furthermore, the HIMALAYA study also suggests that the benefit of immunotherapy combinations may be related to synergistic activation between distinct immune regulatory pathways, pointing to new directions for subsequent research on dual immunotherapy and precision immuno-oncology.

#### 4.2.2. CheckMate 040 Cohort 6

CheckMate 040 Cohort 6 evaluated nivolumab plus cabozantinib with or without ipilimumab in advanced HCC. Median PFS and OS were 5.1 and 20.2 months with the doublet and 4.3 and 22.1 months with the triplet, respectively [118]. Because both regimens contained cabozantinib, these results should not be classified as dual immune checkpoint blockade. In a separate five-year follow-up of nivolumab monotherapy, five-year OS rates were 14% in sorafenib-naive and 12% in sorafenib-experienced cohorts [119].

### 4.3. Locoregional Therapy Combined with Systemic Therapy

#### 4.3.1. Transcatheter Arterial Chemoembolization Combined with Systemic Therapy

Transcatheter arterial chemoembolization (TACE) involves the injection of liquid or particulate embolic agents loaded with chemotherapeutic drugs into the tumor-supplying arteries, reaching the tumor capillaries. This induces tumor ischemia and necrosis while releasing high concentrations of chemotherapeutic agents, thereby promoting tumor cell necrosis and apoptosis, achieving the dual goals of chemoembolization [120,121]. Beyond local tumor control, the mechanism of TACE in HCC also involves activation of innate or adaptive immunity and enhancement of the VEGF pathway, which may synergize with systemic therapy [122].

#### 4.3.2. Early Research Progress

The phase Ib PETAL study evaluated the efficacy and safety of TACE combined with pembrolizumab in HCC [123]. The results showed that the combination did not increase toxicity; the objective response rate (ORR) at 12 weeks post-TACE was 53%, with a median progression-free survival (mPFS) of 8.95 months and a median overall survival (mOS) of 33.5 months. A multicenter retrospective study analyzed the efficacy and safety of TACE combined with atezolizumab plus bevacizumab in intermediate-stage HCC. The ORR was 42.9%, the 1-year PFS rate was 76.2%, and the 1-year OS rate was 90.5% [124].

#### 4.3.3. Confirmatory Study Evidence

In recent years, multiple studies have further advanced the clinical exploration of TACE combined with systemic therapy across different stages of HCC. However, the disease stage, tumor burden, liver function status, and extent of extrahepatic metastasis varied considerably among studies, and the findings should therefore be interpreted within specific clinical contexts.

EMERALD-1 was the first phase III randomized study to report positive results for TACE combined with immunotherapy and anti-angiogenic therapy. The trial primarily enrolled patients with unresectable HCC without extrahepatic metastasis. The results demonstrated that TACE combined with durvalumab plus bevacizumab significantly prolonged median progression-free survival (mPFS) compared with TACE alone (15.0 months vs. 8.2 months) [125]. These findings suggest that the addition of targeted and immunotherapy to locoregional treatment may provide substantial disease-control benefit in patients with predominantly intrahepatic disease.

The LAUNCH trial mainly focused on patients with advanced HCC, including some with portal vein tumor thrombus or high intrahepatic tumor burden. The study showed that TACE combined with lenvatinib significantly improved median overall survival (mOS) compared with lenvatinib monotherapy (17.8 months vs. 11.5 months) [126]. This result indicates that even in advanced-stage disease with high tumor burden, locoregional therapy combined with a tyrosine kinase inhibitor (TKI) may still confer meaningful survival benefit.

The CHANCE 2201 real-world study enrolled 1244 patients with advanced HCC and compared TACE plus targeted-immunotherapy with targeted-immunotherapy alone. The combination strategy achieved superior median overall survival (mOS; 22.6 months vs. 15.9 months), median progression-free survival (mPFS; 9.9 months vs. 7.4 months), and objective response rate (ORR; 41.2%) compared with systemic therapy alone [127]. Nevertheless, given the inherent treatment selection bias and baseline heterogeneity associated with real-world studies, these findings still require validation in prospective randomized trials.

Overall, current evidence suggests that TACE combined with targeted and immunotherapy may have therapeutic potential across different stages of HCC. However, the optimal patient population, treatment timing, sequencing strategy, and long-term survival benefit remain to be further clarified. In particular, for patients with overt extrahepatic metastasis, extremely high tumor burden, or impaired liver function reserve, the actual benefit of locoregional-systemic combination therapy still requires confirmation through additional high-quality prospective studies.

#### 4.3.4. Hepatic Arterial Infusion Chemotherapy Combined with Systemic Therapy

Hepatic arterial infusion chemotherapy (HAIC) involves percutaneous placement of a catheter into the hepatic artery for prolonged, continuous infusion of chemotherapeutic agents under image guidance, thereby increasing local drug concentration in the tumor and enhancing chemotherapeutic efficacy. Current arterial chemotherapy regimens for HCC typically consist of platinum-based and/or fluorouracil-based agents [128,129]. Because HCC is predominantly supplied by the hepatic artery, HAIC has garnered widespread interest in patients with high tumor burden, portal vein tumor thrombus (PVTT), and predominantly intrahepatic progression. In recent years, studies on HAIC combined with targeted therapy and immunotherapy have been increasing.

A multicenter retrospective propensity score matching (PSM) study suggested that compared with HAIC alone, HAIC combined with anti-PD-1 therapy may significantly improve survival outcomes in patients with advanced HCC, with a reported mOS of 40.4 versus 9.7 months and PFS of 22.1 versus 5.7 months. However, the magnitude of the survival benefit should be interpreted cautiously because of the retrospective design, the potential influence of selection bias, heterogeneity in baseline liver function and tumor burden, and differences in subsequent conversion or salvage treatments after initial therapy. In addition, the relatively small sample size may have further amplified the observed survival differences. The prolonged survival observed in the combination group may partly reflect a synergistic interaction between HAIC-induced tumor reduction and immune activation, which could increase the likelihood of conversion therapy and long-term disease control in selected patients [130]. In patients with HCC with a maximum tumor diameter ≥ 8 cm, the HAIC plus atezolizumab plus bevacizumab regimen achieved an objective response rate (ORR) of 74.4%, a disease control rate (DCR) of 93.3%, a median PFS of 6.8 months, and a 1-year OS rate of 60% [131]. Currently, most studies on HAIC combined with systemic therapy remain retrospective analyses or single-arm studies, and patient baseline characteristics, treatment selection, and subsequent therapeutic strategies may all influence the results. In particular, the large variations in OS observed across certain studies should not be simply interpreted as definitive evidence of survival benefit. Furthermore, the efficacy of HAIC may be closely associated with tumor burden, degree of vascular invasion, extrahepatic metastasis status, and liver function reserve. Consequently, the efficacy outcomes show considerable heterogeneity across studies. The optimal patient population, timing of treatment, and long-term survival benefit of HAIC-based combination therapy still require further validation in prospective randomized controlled trials.

#### 4.3.5. Ablation Therapy Combined with Systemic Therapy

Radiofrequency ablation (RFA) uses heat generated by radiofrequency currents to destroy tumor tissue and is an important locoregional therapy for HCC. However, the risk of recurrence after ablation remains high, necessitating the exploration of adjuvant strategies to improve durable disease control. The STORM study showed that sorafenib as adjuvant therapy following resection or RFA for HCC did not improve recurrence-free survival (RFS) [132]. The efficacy of other tyrosine kinase inhibitors combined with RFA in HCC remains to be validated. Based on the mechanism that ablation-induced inflammatory responses and tumor antigen release can activate antitumor immunity, combining PD-1/PD-L1 inhibitors after ablation may enhance local and systemic immune responses. Preliminary data from the phase II NIVOLVE trial showed a median RFS of 26 months in patients with HCC receiving nivolumab after resection or ablation [133]. Several ongoing phase III studies (e.g., CheckMate 9DX, KEYNOTE-937) are evaluating immune checkpoint inhibitors with or without anti-VEGFR agents as adjuvant therapy following surgery or ablation, aiming to provide further clinical evidence supporting this combination strategy.

Microwave ablation (MWA) uses high-frequency electromagnetic waves to generate thermal effects, inducing coagulative necrosis of tumor tissue at high temperatures [134]. MWA has been shown to exert a “1 + 1 > 2” synergistic effect with immunotherapy through mechanisms including enhanced antigen release and presentation, the abscopal effect, and reversal of the immunosuppressive microenvironment [135,136,137]. Duan et al. demonstrated that the combination of MWA and immunotherapy induced non-specific immunity in HCC-bearing mice [138]. Multiple retrospective studies have shown that MWA combined with PD-1 inhibitors effectively prolongs survival in patients with HCC compared with PD-1 inhibitors alone [139,140,141].

Cryoablation uses low-temperature techniques to freeze diseased tissue, achieving inactivation of solid tumors. Compared with high-temperature ablation, the low-temperature environment allows more efficient release of autoantibodies into the bloodstream [142,143]. Gu et al. found that cryoablation combined with dual immune checkpoint blockade enhanced antitumor efficacy in a mouse model of HCC [144]. In the study by Niu et al., cryoablation alone and cryoablation combined with immunotherapy significantly prolonged median overall survival (mOS) in patients with HCC compared with the untreated group and the immunotherapy-alone group (cryoablation plus immunotherapy: 32.0 months; cryoablation alone: 17.5 months; immunotherapy alone: 4.0 months; untreated: 3.0 months) [145].

#### 4.3.6. Radiotherapy Combined with Systemic Therapy

A single-arm phase II study of 17 patients with advanced HCC and portal-vein tumor thrombus evaluated SBRT plus anti-PD-1 therapy. At three months, the reported objective response rate was 100%, and one-year OS and PFS rates were 100% and 84.8%, respectively [146]. These unusually favorable results should be regarded as preliminary because the cohort was small, non-randomized and potentially affected by patient selection and response-assessment methods.

NRG/RTOG 1112 compared sorafenib with SBRT followed by sorafenib in locally advanced HCC; 74% of participants had macrovascular invasion. Median OS was 15.8 versus 12.3 months, but the unadjusted primary analysis did not meet the prespecified significance threshold (HR 0.77; 90% CI 0.59–1.01; one-sided *p* = 0.06). A multivariable analysis adjusted for stratification factors yielded an HR of 0.72 (95% CI 0.52–0.99), and PFS improved from 5.5 to 9.2 months. The trial therefore supports a clinically important signal in selected patients but should not be described as having met its primary OS endpoint [147].

Overall, combination therapy has broadened the therapeutic landscape of HCC, but the magnitude and durability of benefit vary across regimens, disease stages, and study designs. Table 3 and Table 4 summarize the principal combinations, trial identifiers, and reported median overall survival outcomes, enabling structured comparison while underscoring the limitations of cross-trial interpretation. This therapeutic heterogeneity, together with the absence of universal benefit, highlights the need for biomarkers that can match individual patients to the most appropriate treatment strategy, as discussed in the next section.

## 5. Biomarkers and Precision Medicine in HCC

### 5.1. Current Biomarkers and Molecular Stratification

Despite major therapeutic progress, no validated biomarker reliably selects most patients for immune-based systemic therapy in HCC. AFP is currently the most established treatment-selection biomarker because ramucirumab is indicated for patients with AFP ≥ 400 ng/mL after prior sorafenib. Other candidate biomarkers, including PD-L1 expression, tumor mutational burden, Wnt/β-catenin pathway activation, tumour immune signatures, and angiogenic profiles, remain investigational and have not been prospectively validated for routine treatment selection [2,3].

Molecular profiling may identify biologically meaningful subsets, including tumors with FGF19/FGFR4 activation or CTNNB1 alterations. However, intratumour heterogeneity, limited tissue acquisition and the absence of prospectively validated matched therapies currently restrict routine molecularly guided prescribing. Biomarker development should therefore move beyond single analytes toward integrated molecular and clinical models [2].

### 5.2. Liquid Biopsy, Imaging Biomarkers and Multi-Omics

Circulating tumor DNA, circulating tumour cells, plasma proteins, radiomics and multi-omics approaches may enable longitudinal disease monitoring and improve biological stratification. Their current roles are exploratory because analytical platforms, thresholds and clinical endpoints remain heterogeneous. Prospective validation, standardized sampling, and demonstration of treatment-decision utility are necessary before these tools can be incorporated into routine HCC care [148,149].

## 6. Emerging Therapies for HCC

Emerging platforms should be interpreted according to evidence maturity. Most cell therapies, engineered antibodies, gene-editing approaches, oncolytic viruses, and nanocarriers in HCC remain preclinical or in early-phase studies. The subsections below therefore distinguish mechanistic rationale and preliminary activity from evidence sufficient to change routine care.

### 6.1. Chimeric Antigen Receptor T-Cell Therapy

CAR-T therapy redirects genetically modified T cells against cell-surface antigens. In HCC, glypican-3 (GPC3) is the most extensively studied target. Two phase I studies in 13 patients reported two partial responses and one case of stable disease [150,151,152]. A separate phase I study of RUNX3-expressing GPC3-directed CT017 cells, alone or with TKIs, reported manageable safety and preliminary activity in heavily pretreated GPC3-positive HCC [153,154,155,156]. These non-randomized data support continued clinical testing but do not establish efficacy.

Translation is constrained by antigen heterogeneity and escape, limited trafficking, T-cell exhaustion, the immune-tolerant liver microenvironment, and potential on-target/off-tumor toxicity. These limitations provide the rationale for the dual-target, armored and local-delivery strategies discussed below.

### 6.2. CAR-T Combination Strategies

CAR-T activity in solid tumors, including HCC, may be limited by antigen heterogeneity, impaired trafficking and an immunosuppressive microenvironment. Rational strategies under investigation include dual-target CAR designs, cytokine- or checkpoint-armored CAR-T cells, and combination approaches with local therapy or immune-modulating agents. These strategies remain largely preclinical or in early-phase clinical development and should not be considered established treatment options [156,157,158].

### 6.3. TCR-Engineered T-Cell Therapy

T-cell receptor (TCR)-engineered cells can recognize intracellular antigens presented as peptide–human leukocyte antigen complexes, thereby expanding the target repertoire beyond surface antigens. However, their application is constrained by HLA restriction, antigen selection, potential off-target recognition, and manufacturing complexity. Clinical translation of TCR-engineered therapies in HCC remains at an early stage, with current evidence largely limited to early-phase studies and preclinical investigations [159].

### 6.4. NK-Cell Therapy

Natural killer (NK)-cell approaches offer MHC-independent cytotoxicity and may be combined with antibodies, cytokines, or locoregional therapies. Early HCC studies provide biological rationale and preliminary clinical evidence, but optimal cell source, persistence, trafficking, and patient selection remain unresolved [160].

### 6.5. Bispecific Antibodies

Bispecific antibodies may simultaneously engage tumor-associated antigens (TAAs) and immune effector cells or co-target complementary signaling pathways. For HCC, antigen specificity, on-target/off-tumour toxicity and durable activity within an immunosuppressive tumour microenvironment remain major developmental challenges. Available evidence is predominantly preclinical or derived from early-phase clinical studies, and the clinical efficacy of bispecific antibody-based strategies in HCC remains to be established [161].

### 6.6. Antibody–Drug Conjugates

Antibody–drug conjugates (ADCs) provide a strategy to selectively deliver cytotoxic payloads to tumor-associated antigens through antibody-mediated targeting and intracellular payload release. ADC development in HCC remains at an early stage, and therapeutic success will depend on antigen expression uniformity, efficient internalization, payload selection, and safety considerations in the context of chronic liver disease. Further clinical investigation is required to determine whether ADCs can achieve meaningful therapeutic benefit in molecularly selected HCC populations [162].

### 6.7. Therapeutic Cancer Vaccines

Peptide, dendritic-cell, viral-vector, and messenger RNA vaccine strategies aim to broaden or restore antitumor immunity by enhancing tumour antigen recognition and T-cell activation. Their major translational challenges include overcoming HCC-associated immune suppression and tolerance, as well as achieving sufficient immunogenicity in the context of chronic liver disease. Combination strategies with immune checkpoint blockade or locoregional therapies are biologically attractive but remain investigational and require clinical validation [163].

### 6.8. CRISPR-Based Approaches

CRISPR-based genome editing is being investigated in HCC as a research platform for defining driver pathways and as an enabling technology for adoptive cell therapy. Potential applications include disrupting inhibitory receptors in T or NK cells, improving persistence or trafficking, editing tumor-resistance pathways, and engineering viral vectors. Most HCC evidence remains preclinical, and off-target editing, genotoxicity, delivery, immune responses, and manufacturing control remain unresolved [159].

The most controllable near-term application may be ex vivo editing of adoptive immune cells, because edited products can be characterized before infusion. Clinical translation will require HCC-specific target validation, standardized off-target assessment, long-term genomic safety surveillance, and early-phase trials with explicit stopping rules. CRISPR-based approaches should therefore be presented as experimental enabling technologies rather than established HCC treatments [159].

### 6.9. Tumor-Infiltrating Lymphocyte Therapy

Tumor-infiltrating lymphocytes (TILs) are a subset of lymphocytes present within tumor tissue that can recognize and specifically kill tumor cells. The basic principle of TIL therapy involves isolating TILs from a patient’s tumor tissue, expanding them ex vivo, and then reinfusing them into the patient to enhance the ability of the patient’s own immune system to recognize and eliminate tumor cells, thereby achieving therapeutic antitumor effects [164]. Results from a Chinese clinical trial of autologous TIL therapy combined with a PD-1 inhibitor in advanced HCC showed that two patients—one with HCC and bilateral adrenal metastases and another with HCC and bilateral lung metastases—benefited from TIL therapy. In the patient with bilateral lung metastases, after 11 weeks of treatment with the TIL plus PD-1 monoclonal antibody regimen, the lung metastatic lesions disappeared, and a complete response (CR) was achieved, suggesting the potential of TIL therapy combined with immunotherapy in the treatment of HCC [165].

Clinical evidence for TIL therapy in HCC is limited to highly selected cases and small studies and cannot establish durable efficacy. Manufacturing complexity, cost, variable TIL yield, lymphocyte exhaustion, and the immunosuppressive liver microenvironment remain important constraints. Gene editing and combinations with local or systemic therapies are hypotheses for early-phase testing, not established routes to long-term survival [164,165].

### 6.10. Oncolytic Virus Therapy

Oncolytic viruses can lyse tumor cells and stimulate antitumour immunity [166]. NDV-GT showed activity in a CRISPR-engineered primate HCC model; a first-in-human study enrolled 20 patients with different recurrent or metastatic cancers and reported a disease control rate of 90% without serious adverse events [167]. Because the cohort was small and tumour types were mixed, these findings establish feasibility but not HCC-specific efficacy. A multicentre phase I trial of VG161 in refractory HCC reported tolerability and preliminary activity, but efficacy remains to be confirmed [168].

Clinical efficacy of systemic oncolytic virotherapy in HCC remains unproven. Neutralization in the circulation, limited intratumoral delivery, vector manufacturing, and long-term safety are key constraints. Future studies should define tumour-specific activity, dose, route, and combination strategy in adequately controlled HCC cohorts rather than assume that early feasibility signals will translate into phase III benefit.

### 6.11. Nano-Drug Delivery Systems

Nanocarriers such as liposomes, exosomes, and polymeric or metal-based nanoparticles can alter drug exposure and distribution [169]. In experimental HCC models, N-acetylgalactosamine-modified lipid nanoparticles co-delivering a doxorubicin prodrug and sorafenib increased cellular uptake and tumor suppression relative to unmodified carriers [170]. HCC-derived exosomes engineered with HMGN1 also enhanced dendritic-cell-mediated T-cell activation in a preclinical system [171]. These studies support mechanistic feasibility but do not establish clinical benefit.

Most actively targeted or environment-responsive nanoplatforms remain preclinical or in early clinical development. Hepatic clearance, variable tumor penetration, long-term biodistribution, and scalable manufacturing remain unresolved. HCC-specific translation will require reproducible pharmacokinetics, a validated delivery advantage and comparative safety before combinations with immunotherapy, gene editing, or locoregional treatment can be justified [169,170,171].

## 7. Conclusions

The systemic therapy landscape for unresectable HCC has undergone a paradigm shift, evolving from single-agent tyrosine kinase inhibition to immune-based combination regimens as the preferred first-line strategy. Phase III evidence from IMbrave150, HIMALAYA, ORIENT-32, and HEPATORCH has established anti-VEGF plus anti-PD-1/PD-L1 combinations and dual immune checkpoint blockade as validated first-line options, while multikinase inhibitors retain a defined role in patients with contraindications to immunotherapy or anti-angiogenic therapy. No single regimen is universally optimal; treatment selection must integrate tumor stage, liver functional reserve, performance status, bleeding and autoimmune risk, etiological background, regional drug availability, and patient preferences. The transition from locoregional to systemic therapy in intermediate-stage disease and the rational sequencing of TKIs after first-line immunotherapy failure represent critical decision points that currently lack prospective randomized evidence and must therefore be individualized.

Molecular pathway analysis reveals a clear hierarchy of therapeutic relevance. VEGF/VEGFR signaling is the only axis with validated clinical utility through approved multikinase inhibitors and anti-angiogenic combinations, whereas direct targeting of the RAS/RAF/MEK/ERK, PI3K/AKT/mTOR, and JAK/STAT pathways has not yielded a standard HCC therapy despite compelling biological rationale and preclinical activity. The Wnt/β-catenin pathway, though not yet druggable, has emerged as a critical predictor of immune exclusion and primary resistance to checkpoint inhibition, highlighting the convergence of oncogenic signaling and tumor immunology. This evidence hierarchy underscores that pathway complexity, adaptive resistance, and extensive crosstalk necessitate biomarker-guided combination strategies rather than single-axis inhibition.

Biomarker development remains the most pressing unmet need in HCC precision medicine. AFP ≥ 400 ng/mL for ramucirumab selection is the sole validated treatment-stratification biomarker. Candidate biomarkers—including PD-L1 expression, tumor mutational burden, FGF19/FGFR4 activation, CTNNB1 alterations, liquid-biopsy signatures, radiomic features, and multi-omics classifications—remain investigational and require prospective analytical and clinical validation before routine implementation. Future biomarker research should progress from single-analyte approaches toward integrated molecular-clinical models that incorporate tumor biology, liver function, and host factors to enable truly precision-guided treatment selection.

Emerging therapeutic platforms—including CAR-T and TCR-engineered T-cell therapy, NK-cell approaches, bispecific antibodies, antibody–drug conjugates, therapeutic vaccines, CRISPR-based gene editing, oncolytic viruses, and nanocarrier delivery systems—provide mechanistically promising but clinically unproven strategies. Each platform faces HCC-specific translational barriers: antigen heterogeneity, impaired T-cell trafficking and persistence, the immunosuppressive liver microenvironment, delivery limitations, and safety concerns in the context of chronic liver disease. These approaches should be regarded as testable hypotheses requiring rigorous early-phase evaluation with predefined efficacy and safety thresholds, not as near-term treatment options.

Looking forward, the field must prioritize several interconnected objectives: prospective sequencing studies to define optimal post-immunotherapy strategies; biomarker-enriched trial designs that match molecular subtypes to targeted interventions; rigorously controlled early-phase studies of emerging platforms with explicit stopping rules; and standardized integration of locoregional and systemic therapies across disease stages. Sustained progress will depend on maintaining fidelity between claim strength and evidence maturity, bridging molecular discovery with clinical validation, and embedding liver function and patient-centered outcomes into the framework of precision oncology for HCC.

## Figures and Tables

**Table 1:** Practical BCLC-based treatment considerations and sequencing principles for HCC.

Clinical Context	Preferred Treatment Consideration	Key Restriction or Caveat
BCLC 0/A	Assessment for resection, local ablation or liver transplantation.	Treatment selection depends on tumor number, size and location, portal hypertension, liver functional reserve and transplant eligibility.
BCLC B	Selective TACE for appropriate candidates; transplantation or upfront systemic therapy may be considered in selected patients; reassess after each treatment.	Avoid repeated ineffective TACE. Transition to systemic therapy when TACE is unsuitable, unlikely to provide meaningful benefit, or when TACE refractoriness/failure develops.
BCLC C, eligible for immune-based combination therapy	Atezolizumab plus bevacizumab or the STRIDE regimen; other approved immune-based combinations may be considered according to regional guidance and availability.	For atezolizumab–bevacizumab, assess variceal and other bleeding risks and contraindications to anti-VEGF therapy. For all ICI-based regimens, consider autoimmune disease, prior solid-organ transplantation, liver reserve and immune-related toxicity risk.
BCLC C, unsuitable for ICI-based therapy	TKI-based therapy, usually lenvatinib or sorafenib.	Select according to liver function, contraindications, anticipated tolerability, drug interactions and regional availability.
After disease progression	Subsequent systemic therapy based on prior treatment, liver function, toxicity profile, AFP level and drug availability; clinical-trial enrolment is encouraged.	No single optimal sequence after first-line ICI-based therapy has been established in prospective randomized trials.
BCLC D	Best supportive and palliative care, symptom management and goals-of-care discussions.	Anticancer treatment is generally inappropriate unless poor clinical status is caused by a potentially reversible tumor-related complication or an exceptional treatment opportunity exists.

**Table 2:** Targeted monotherapies and overall survival in HCC.

Drug	Study Name/NCT ID	Target	Control Group	Experimental Group mOS	Control Group mOS
Sorafenib	SHARP [83]	Multi-target	Placebo	10.7	7.9
Lenvatinib	REFLECT [84]	Multi-target	Sorafenib	13.6	12.3
Donafenib	ZGDH3 [86]	Multi-target	Sorafenib	12.1	10.3
Regorafenib	RESORCE [14]	Multi-target	Placebo	10.6	7.8
Ramucirumab	REACH-2 [91]	VEGFR-2	Placebo	8.5	7.3
Tivantinib	NCT01755767 [95]	c-Met	Placebo	8.4	9.1
Cabozantinib	CELESTIAL [96]	Multi-target	Placebo	10.2	8
Brivanib	BRISK-FL [103]	VEGFR, PDGFR, FGFR	Sorafenib	9.5	9.9
Everolimus	EVOLVE-1 [109]	mTOR	Placebo	7.6	7.3

**Table 3:** Combination regimens and trial identifiers in HCC.

Combination Therapy			Study Name/NCT ID
Atezolizumab	Bevacizumab		IMbrave150 [89]
Sintilimab	Bevacizumab biosimilar IBI305		ORIENT-32 [112]
Pembrolizumab	Lenvatinib		KEYNOTE-524 (Phase Ib) [113]
Pembrolizumab	Lenvatinib		LEAP-002 [114]
Cabozantinib	Atezolizumab		COSMIC-312 [115]
Durvalumab	Tremelimumab		HIMALAYA [117]
Nivolumab	Cabozantinib	±Ipilimumab	CheckMate 040 (Cohort 6) [118]
TACE	Pembrolizumab		PETAL (Phase Ib) [123]
TACE	Durvalumab	Bevacizumab	EMERALD-1 [125]
TACE	Lenvatinib		LAUNCH [126]
TACE	ICI	anti-VEGF antibody/TKI	CHANCE 2201 [127]
SBRT	Sorafenib		RTOG 1112 [147]

**Table 4:** Comparison of median overall survival (mOS) in major clinical trials of combination therapy.

Study Name/NCT ID	Control Group	Experimental Group mOS	Control Group mOS
IMbrave150 [89]	Sorafenib	19.2	13.4
ORIENT-32 [112]	Sorafenib	Not Reached	10.4
KEYNOTE-524 (Phase Ib) [113]	None (single-arm)	22	Not applicable
LEAP-002 [114]	Lenvatinib + Placebo	21.2	19
COSMIC-312 [115]	Sorafenib	15.4	15.5
HIMALAYA [117]	Sorafenib	16.4	13.8
CheckMate 040 (Cohort 6) [118]	None (single-arm)	20.2 (doublet); 22.1 (triplet)	Not applicable
PETAL (Phase Ib) [123]	None (single-arm)	33.5	Not applicable
EMERALD-1 [125]	TACE + Placebo	Not Reached	Not Reached
LAUNCH [126]	Lenvatinib	17.8	11.5
CHANCE 2201 [127]	ICI + anti-VEGF antibody/TKI	22.6	15.9
RTOG 1112 [147]	Sorafenib	15.8	12.3

## Data Availability

No new data were created or analyzed in this study. Data sharing is not applicable to this article.

## Abbreviations

The following abbreviations are used in this manuscript:

HCC	Hepatocellular Carcinoma
VEGF	Vascular Endothelial Growth Factor
VEGFR	Vascular Endothelial Growth Factor Receptor
FGFR	Fibroblast Growth Factor Receptor
c-MET	Hepatocyte Growth Factor Receptor (HGFR)
PI3K	Phosphoinositide 3-Kinase
AKT	Protein Kinase B
mTOR	Mammalian Target of Rapamycin
JAK	Janus Kinase
STAT	Signal Transducer and Activator of Transcription
TKI	Tyrosine Kinase Inhibitor
ORR	Objective Response Rate
ICI	Immune Checkpoint Inhibitor
PD-1	Programmed Cell Death Protein 1
PD-L1	Programmed Death-Ligand 1
RTK	Receptor Tyrosine Kinase
MAPK	Mitogen-Activated Protein Kinase
EGFR	Epidermal Growth Factor Receptor
PDGFR	Platelet-Derived Growth Factor Receptor
IGFR	Insulin-Like Growth Factor Receptor
EMT	Epithelial–Mesenchymal Transition
HGF	Hepatocyte Growth Factor
FGF	Fibroblast Growth Factor
RAF	Rapidly Accelerated Fibrosarcoma Kinase
MEK	Mitogen-Activated Protein Kinase
ERK	Extracellular Signal-Regulated Kinase
RAS	Rat Sarcoma Virus Oncogene
mTORC1	Mechanistic Target of Rapamycin Complex 1
mTORC2	Mechanistic Target of Rapamycin Complex 2
HIF-2α	Hypoxia-Inducible Factor 2-Alpha
PDK1	3-Phosphoinositide-Dependent Protein Kinase 1
SOCS5	Suppressor of Cytokine Signaling 5
SRPK2	Serine/Arginine-Rich Protein Kinase 2
SREBP-1	Sterol Regulatory Element-Binding Protein 1
CTNNB1	Catenin Beta 1 Gene
TCF	T-Cell Factor
LRP	Low-Density Lipoprotein Receptor-Related Protein
GSK-3β	Glycogen Synthase Kinase 3 Beta
APC	Adenomatous Polyposis Coli
CK1α	Casein Kinase 1 Alpha
CYP2E1	Cytochrome P450 2E1
TYK2	Tyrosine Kinase 2
AFP	Alpha-Fetoprotein
mOS	Median Overall Survival
mPFS	Median Progression-Free Survival
mTTP	Median Time to Progression
DCR	Disease Control Rate
DOR	Duration of Response
HBV	Hepatitis B Virus
CTLA-4	Cytotoxic T-Lymphocyte-Associated Protein 4
TACE	Transarterial Chemoembolization
HAIC	Hepatic Arterial Infusion Chemotherapy
RFA	Radiofrequency Ablation
MWA	Microwave Ablation
SBRT	Stereotactic Body Radiation Therapy
CAR-T	Chimeric Antigen Receptor T-Cell
TIL	Tumor-Infiltrating Lymphocyte
GPC3	Glypican-3
RUNX3	Runt-Related Transcription Factor 3
CRISPR	Clustered Regularly Interspaced Short Palindromic Repeats
α1,3-GT	Porcine α1,3-Galactosyltransferase
NDV-GT	Newcastle Disease Virus Expressing α1,3-GT
LNP	Lipid Nanoparticle
NAcGal	N-Acetylgalactosamine
HMGN1	High-Mobility Group Nucleosome-Binding Domain 1
